# Recent evidence that TADs and chromatin loops are dynamic structures

**DOI:** 10.1080/19491034.2017.1389365

**Published:** 2017-12-14

**Authors:** Anders S. Hansen, Claudia Cattoglio, Xavier Darzacq, Robert Tjian

**Affiliations:** a Department of Molecular and Cell Biology, Li Ka Shing Center for Biomedical and Health Sciences, CIRM Center of Excellence, University of California, Berkeley, CA, USA; b Howard Hughes Medical Institute, Berkeley, CA, USA

**Keywords:** CTCF, cohesin, 3D genome, single-molecule imaging, dynamics, FRAP, topological domains, chromatin loops, loop extrusion, modeling

## Abstract

Mammalian genomes are folded into spatial domains, which regulate gene expression by modulating enhancer-promoter contacts. Here, we review recent studies on the structure and function of Topologically Associating Domains (TADs) and chromatin loops. We discuss how loop extrusion models can explain TAD formation and evidence that TADs are formed by the ring-shaped protein complex, cohesin, and that TAD boundaries are established by the DNA-binding protein, CTCF. We discuss our recent genomic, biochemical and single-molecule imaging studies on CTCF and cohesin, which suggest that TADs and chromatin loops are dynamic structures. We highlight complementary polymer simulation studies and Hi-C studies employing acute depletion of CTCF and cohesin, which also support such a dynamic model. We discuss the limitations of each approach and conclude that in aggregate the available evidence argues against stable loops and supports a model where TADs are dynamic structures that continually form and break throughout the cell cycle.

## Introduction

**Figure 1.**
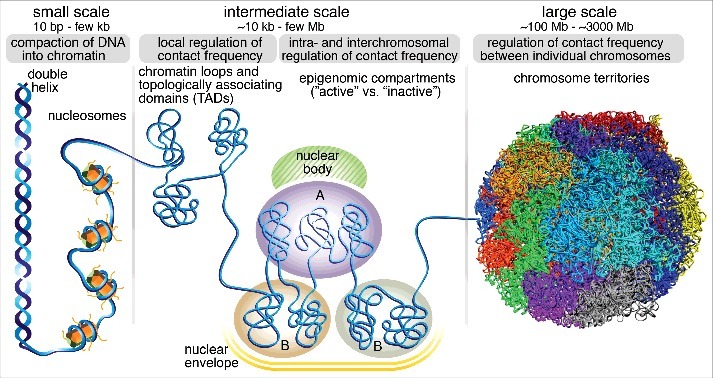
Chromosome structure and function is organized at multiple scales. At the smallest scale, DNA is folded into a double helix, which gets compacted into ∼11 nm nucleosomes, whereby 147 bp of DNA wrap around a histone octamer. Functionally, nucleosomes regulate access of DNA-binding proteins and serve as modules for epigenetic modifications, which regulate gene expression. At the intermediate scale of tens of kilobases to a few megabases, chromatin is organized into Topologically Associating Domains (TADs) with a median size of a few hundred kilobases. Functionally, TADs are characterized by preferential contact of loci within them, and critically control enhancer-promoter interactions, and relative insulation from adjacent TADs. At a similar scale of TADs, chromatin is also organized into epigenomic A/B “compartments”, whereby active chromatin (A) tends to contact with other segments of active chromatin and localize in proximity of certain nuclear bodies such as nuclear speckles, while inactive chromatin (B) tends to contact with inactive chromatin and to be associated with the nuclear lamina. At the largest scale, particular chromosomes tend to associate with other chromosomes and form stereotyped chromosome territories inside the cell nucleus. The image used to illustrate chromosome territories was generously provided by Stevens *et al.*^76^

Mammalian genomes are folded at multiple scales and each scale highlights an important interplay between structure and function^1,2^ (Fig. 1). At the smallest scale, DNA is compacted into 11-nm nucleosomes, where 147 bases of DNA wrap around an octamer of histone proteins. Nucleosomes function structurally to compact DNA into chromatin, but also impact gene regulation by controlling accessibility to DNA binding proteins and function as modules for epigenetic inheritance. At the intermediate scale of tens of kilobases to a few megabases, chromatin is organized into spatial domains (Fig. 1). First, chromatin is organized into Topological Associating Domains (TADs) that are characterized by preferential contacts between loci inside the same TAD and insulation from loci in adjacent TADs.^3,4^ Thus, TADs likely regulate enhancer-promoter contacts and gene expression. Second, at a similar scale of tens of kilobases to a few megabases, regions of similar epigenomic states tend to contact each other to form so-called A and B compartments^5^ composed of largely “active” and “inactive” chromatin, respectively. Interestingly, rather than one structural feature serving as the building block of the other, TADs and A/B compartments appear to be formed by distinct mechanisms. Whereas TADs are strictly local, epigenomic compartments form both intra- and interchromosomal contacts with other compartments of the same state, giving rise to a “plaid” pattern. Finally, at the largest scale, chromosomes are organized into chromosome territories,^6^ whereby particular chromosomes adopt stereotyped positions in the nucleus (Fig. 1).

This Extra View article focuses on the intermediate scale of chromatin organization in mammalian cells. First, we review recent studies that have enriched our understanding of the nature and function of TADs and epigenomic compartments. We then discuss recent studies establishing the causal roles of CCCTC-binding factor (CTCF) and cohesin in the formation and maintenance of TADs. In particular, we focus on the question of whether TADs and chromatin loops are stable or dynamic structures. We integrate our recent imaging studies on CTCF and cohesin^7^ suggesting that TADs are dynamic, with complementary evidence from polymer simulation studies^8,9^ and recent Hi-C studies employing acute depletion of CTCF and cohesin subunits and regulators.^10–15^ We conclude with a critical discussion of the advantages and limitations of each approach and highlight key future directions.

## TADs and epigenomic compartments: Interplay between structure and function

In mammalian nuclei, active chromatin tends to associate with active chromatin and vice versa giving rise to a “plaid” pattern in maps obtained by genome-wide chromosome conformation capture studies (Hi-C).^5^ These epigenomic compartments range in size from 6 kb to more than 5 Mb.^16^ The mechanisms through which epigenomic compartments are formed are not well understood, though compartments composed of mainly active chromatin tend to be enriched near nuclear speckles away from the nuclear envelope.^17^ In contrast, compartments composed of repressed chromatin tend to be located near the nuclear envelope through lamina association and are also referred to as Lamina-Associated Domains (LADs)^17^ (Fig. 1). To which extent function (e.g. gene expression state) drives compartmentalization and to which extent compartmentalization affects function is not currently clear and neither is the full role of nuclear bodies in compartmentalization.

**Figure 2.**
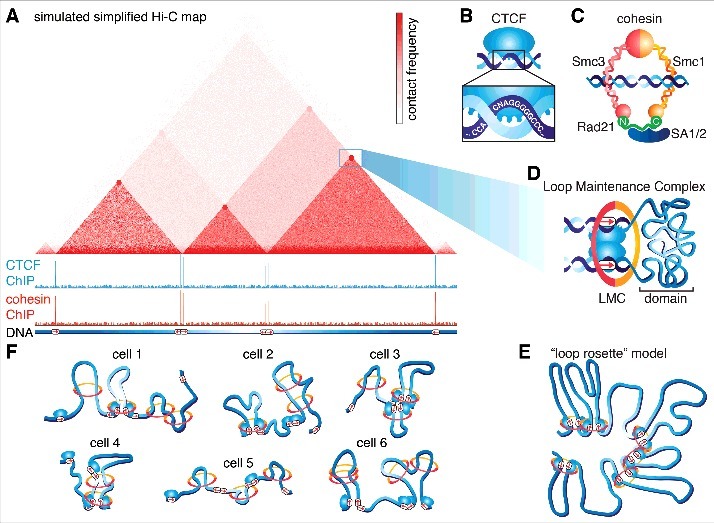
TADs, chromatin loops and the role of CTCF and cohesin. (A) A simulated and simplified Hi-C map. The color scale corresponds to contact frequency (darker red, more frequent contacts). TADs appear as triangles, within which there are more frequent chromatin contacts and are often marked by “cornerpeaks”, suggesting that they are held together by a chromatin loop. Below, simulated CTCF and cohesin ChIP-Seq tracks illustrating that TAD and loop boundaries are almost always bound by CTCF and cohesin. At the bottom, DNA with the location and orientation of CTCF binding sites listed (red arrows denote CTCF binding site orientation). Note that Hi-C and ChIP-Seq data in this sketch is simulated and simplified. This sketch was inspired Fig 2a in Merkenschlager and Nora.^1^ (B) Sketch of CCCTC-binding factor, CTCF, an 11-zinc finger DNA binding protein with its consensus DNA binding sequence shown. (C) Sketch of the cohesin complex composed of the proteins Smc1, Smc3 and Rad21, which closes the ring, and the SA1/2 subunit which is involved in protein interactions. Cohesin topologically entraps chromatin within its lumen. Please note that whether cohesin functions as a single ring or a pair of rings remains a subject of debate. (D) The presence of CTCF and cohesin bound “corner peaks” in Hi-C maps are generally assumed to correspond to a chromatin loop held together by CTCF and cohesin as sketched. We refer to this protein complex holding together a loop as a Loop Maintenance Complex (LMC). (E) “Loop rosette” model, where TADs are held together by loops and TADs without cornerpeaks form passively from adjacent loop domains. This sketch was inspired by Fig 6F in Rao *et al..*^16^ (F) TADs may emerge at the population level when averaged over many heterogeneous single-cell genome topologies. Cohesin is sketched as rings and CTCF as in (B). This picture assumes that TADs are formed by cohesin-mediated extrusion, which stops at occupied CTCF binding sites. This sketch was inspired by Fig 7A in Fudenberg *et al.*^8^ Panels (B-D) have been adapted and reproduced from Hansen *et al.*^7^

Unlike epigenomic compartments, TADs are not defined by chromatin state but by an elevated frequency of contacts within them and reduced contacts to loci inside adjacent TADs. TADs were identified through Hi-C^3,4^ and their existence have since been confirmed by alternative methods such as intrinsic 3C (i3C),^18^ which avoids the cross-linking step and Genome Architecture Mapping (GAM),^19^ which avoids the ligation step. TADs are somewhat conserved between different cell types and different species.^4,20^ Moreover, TADs often have a nested, hierarchical configuration, where smaller TADs can make up larger TADs, and range in size from ∼40 kb to ∼3 MB with a median size of ∼185 kb^16^, though these numbers depend somewhat on the resolution of the Hi-C data used to identify them (Fig. 2A). A large body of evidence suggests that TADs correspond to functional regulatory domains by primarily restricting contact between enhancers and promoters to occur within the same TAD^3,21–25^ and physical contact between enhancer-promoter pairs appears to be sufficient for gene activation.^26^ Accordingly, deletion of a boundary between two TADs causes ectopic contacts between previously insulated loci^3^ and can cause aberrant gene expression through improper enhancer-promoter contact.^27,28^ Even though two loci only contact each other ∼2-fold more frequently if they are within the same TAD instead of in different TADs,^29^ loss of a TAD boundary between limb enhancers and the *wnt6* gene is sufficient to upregulate *wnt6* expression and cause developmental defects in both mice and humans.^28^ Thus, enhancers appear to have limited intrinsic specificity for their target promoters. Instead, by regulating contact probability, TADs appear to be one of the determinants of functional and specific enhancer-promoter interactions.^29^ Due to their crucial role in regulating gene expression^1^ and other biological processes such as replication timing,^30^ understanding the mechanisms through which TADs are formed and maintained has been the subject of intense interest.

## TADs are formed by cohesin and TAD boundaries are defined by CTCF

After TADs were discovered through Hi-C studies, it quickly became clear that TAD boundaries are near universally enriched in CTCF and cohesin ChIP-Seq peaks,^3,4,16^ suggesting an important functional role of CTCF and cohesin in TAD formation and maintenance (Fig. 2A). Classically described as an “insulator”, CTCF is an 11-zinc finger DNA binding protein (Fig. 2B)^31,32^ with a central zinc-finger domain flanked by large C- and N-terminal domains that appear to be unstructured and whose function is poorly understood.^33^ Cohesin is a ring-shaped multi-protein complex composed of Smc1, Smc3, Rad21 and SA1/2. Cohesin is thought to topologically entrap DNA (Fig. 2C),^34^ a structural feature that enables cohesin to mediate multiple functions including sister chromatid cohesion and homologous recombination.^35^ Many TADs display prominent “corner peaks” in Hi-C maps, indicating a strong contact between the CTCF- and cohesin-bound TAD boundaries. This readily suggested a model wherein CTCF binds its cognate sites and recruits cohesin, which then folds the in-between chromatin into a loop structure. TADs with a strong corner peak are also referred to as “loop domains”^16^ and we recently referred to the protein complex hypothesized to hold the loop together as a Loop Maintenance Complex (LMC; Fig. 2D).^7^ Notably, TADs without strong corner peaks tend to be flanked by loop domains suggesting that they may emerge passively from constraints exerted by neighboring loops^16^ or by transitions in compartment state (e.g. active to inactive).^16^ Nevertheless, this picture of adjacent, well-ordered TADs is very likely too simplistic (“loop rosette” or “looped florets”; Fig. 2E). Both DNA FISH^3,9,36–39^ and single-cell Hi-C^40,41^ studies show that genome organization is highly variable between cells and that even strong Hi-C loop anchors only contact in a small subset of cells, arguing that a simultaneous “loop rosette” occurs very rarely in a single cell.

How are TADs formed? Since almost all TADs are demarcated by CTCF ChIP-Seq peaks,^16^ but only around one-third of CTCF ChIP-Seq peaks flank TADs,^1,7^ it was unclear what role CTCF played. A key piece of the puzzle came from the “convergent rule”: loop-anchored TADs almost always form between CTCF binding sites in a convergent orientation.^16,20^ Since cohesin can remain topologically engaged and slide on both naked DNA^42–44^ and chromatin,^45,46^ this favors a linear tracking mechanism,^47^ known as the “loop extrusion model”.^8,9,48^ In this model, cohesin spontaneously engages chromatin and begins extruding a DNA loop. Loop extrusion then continues until cohesin spontaneously falls off or until it encounters an occupied CTCF binding site in the convergent orientation. Since the orientation of the CTCF binding site determines the orientation of the CTCF protein, this may explain how an extruding cohesin complex distinguishes between convergent and divergent CTCF binding sites. Consistently, cohesin subunit ChIP-Seq peaks tend to be inside the loop relative to CTCF ChIP-Seq peaks.^8,9^ Although the loop extrusion model remains a hypothesis, it has proved remarkably resilient to testing. First, polymer simulations of the extrusion model can quantitatively explain high-resolution Hi-C data.^8,9^ Second, it can nicely explain why deletion of CTCF binding sites can cause merging of TADs and how inversion of CTCF binding sites can either create new boundaries or cause merging depending on context as has been observed experimentally.^9,49–51^ Third, although it requires cohesin extrusion to be quite fast, closely related SMC-condensin complexes were recently found to extrude at ∼50 kb/min in *B. subtilis*^52^ and ∼16-19 kb/min in *Caulobacter.*^53^ Finally, ∼18% of TAD boundaries appear to be partially resistant to CTCF loss,^10^ suggesting that additional boundary factors, such as active promoters^4^ since engaged RNA Pol II can block cohesin extrusion^46^ as well as alternative architectural factors, may also play a role. However, as long as these can block cohesin extrusion, this remains consistent with the extrusion hypothesis.

As clearly articulated by Fudenberg and Imakaev *et al.*,^8^ the picture that emerges from the loop extrusion model is quite different from the “loop rosette” model (Fig. 2E). Instead, TADs emerge at the population-level when averaging over many loop extrusion complexes positioned differently in single cells (Fig. 2F).^8,9^ The CTCF/cohesin anchored loop domain matching the Hi-C corner-peak actually only exists in a small subset of cells, which is also consistent with the high levels of cell-to-cell variability in contact probability observed in DNA FISH studies.^3,9,36–39^ Moreover, the emerging roles of CTCF and cohesin are quite different. Using slightly anthropomorphic imagery, CTCF is the “passive” partner and predominantly sits bound to its cognate sites and “waits” for extruding cohesin complexes, whereas cohesin plays the “active” role by constantly moving along and extruding DNA until it “finds” CTCF. This picture also helps explain recent studies employing acute degradation of CTCF or cohesin, which has been challenging to achieve experimentally since both proteins are essential. In accordance with the extrusion model, TADs and well-defined Hi-C loops largely disappear when either CTCF^10,14^ or cohesin^11,13–15^ is strongly depleted, but for different reasons. CTCF loss causes TADs to disappear because cohesin extrusion no longer stops at convergent CTCF binding sites. Thus, chromatin is still compacted and extruded by cohesin, but without sharp boundaries, TADs disappear in Hi-C maps.^10,14^ In contrast, cohesin loss leads to disappearance of TADs because extrusion no longer takes place.^11,13–15^ Cohesin loss has no apparent effect on CTCF binding to chromatin,^13^ consistent with CTCF functioning upstream of cohesin. Consistent with their different roles, CTCF loss has essentially no effect on genome-averaged contact probability at any distance scale,^10^ whereas cohesin loss significantly reduces contact probability at the scale of tens to hundreds of kilobases,^11^ i.e. the typical scale of loci within the same TAD. Likewise, CTCF loss has only a small effect on epigenomic compartments,^10^ whereas cohesin loss increases the degree of epigenomic compartmentalization ∼2-fold.^11,13^ This result also argues against a hierarchical model where TADs are the building blocks of compartments and demonstrates that TADs and epigenomic compartments are formed by distinct and mutually antagonistic mechanisms.

In conclusion, recent studies^10–15^ employing induced CTCF and cohesin degradation now strongly support a model where cohesin forms TADs and CTCF establishes TAD boundaries. However, the question of how TADs are maintained remains. Specifically, are TADs and chromatin loops stable or dynamic structures?

## Are TADs and chromatin loops dynamic or stable structures?

We will now focus our attention on the question of whether TADs and chromatin loops are stable or dynamic structures. Although answering this question is seminal to understanding the function of TADs, the issue has remained unclear largely because of technical challenges. Since Hi-C provides a static snapshot derived from the average of a large cell population, Hi-C based methods are necessarily limited in their ability to reveal dynamics and heterogeneity. While single-cell methods such as single-cell Hi-C and DNA-FISH can report on cell-to-cell variability, they still provide a terminal snapshot and thus can only give limited dynamic information. What has been clear is that TADs and loops are absent in mitosis, but present in G1-phase.^54,55^ Thus, TADs form and dissolve at least once every cell cycle in cycling mammalian cells (∼15-30 hours). Perhaps because TADs and loops are so clearly and reproducibly visible in Hi-C maps, several studies have inferred that TADs must indeed be very stable structures.^32,56–58^ However, here we will put forward the view that TADs and chromatin loops are instead quite dynamic structures that constantly form and break at a time-scale of several minutes to tens of minutes in typical mammalian cells. We will integrate and consolidate the evidence from three orthogonal approaches and critically review the strengths and weaknesses of each them.

## Single-molecule imaging approaches suggest that TADs and loops are dynamic

We recently used live-cell imaging to study whether TADs and chromatin loops are dynamic.^7^ A unique advantage of live-cell imaging is that single protein molecules can be followed over time and their dynamics can thus be directly observed at the single-cell level. Since multiple studies show that TADs and chromatin loops disappear without CTCF^10,14^ and cohesin,^11,13–15^ we reasoned that the most stable protein of CTCF and cohesin should set an upper limit on the stability of TADs and chromatin loops. To study the dynamics of CTCF and cohesin, we used CRISPR/Cas9-mediated genome-editing in mouse embryonic stem cells (mES cells) and human U2OS cells to homozygously tag both proteins with HaloTag or SNAP_f_-Tag, which allows covalent conjugation to bright Janelia Fluor (JF) dyes suitable for single-molecule imaging.^59,60^ First, we performed a series of control experiments to verify that tagging CTCF and cohesin – both essential proteins – did not affect their function or expression levels. We additionally performed ChIP-Seq and found that although CTCF binds ∼2-fold more sites than cohesin (68,077 vs. 33,434 called peaks; note that cohesin extrusion likely “spreads out” its ChIP signal and thus results in fewer “peaks”), essentially all cohesin ChIP-Seq peaks co-localize with CTCF peaks. We next performed co-immunoprecipitation (IP) experiments and found that CTCF IP pulled down all the subunits of the cohesin complex consistent with earlier reports,^61^ demonstrating that CTCF and cohesin form a biochemically stable protein complex, which is not affected by endogenous tagging. In summary, CTCF and cohesin are required for chromatin looping,^10–15^ form a biochemically stable protein-complex and both bind chromatin at loop anchors. Thus, the simplest interpretation is that CTCF and cohesin form a complex on chromatin which holds together chromatin loops and we refer to this population as a Loop Maintenance Complex (LMC; Fig. 2D).

Since the dynamics of the LMC should reflect the dynamics of TADs and chromatin loops, we next studied the chromatin binding dynamics of CTCF and cohesin. First, we used single-molecule tracking (SMT) to follow the binding dynamics of single Halo-CTCF molecules in mES and U2OS cells. Careful optimization of imaging conditions in combination with the bright JF-dyes^60^ made it possible to follow individual binding events from less than a second (non-specific binding) up to rare events lasting ∼10 minutes. After correcting for photo-bleaching, drift etc. we found an average CTCF SMT residence time of ∼1 min in mES cells for specific binding events at cognate sites and somewhat longer in U2OS cells. Next, to cross-validate this observation using an orthogonal technique, we performed Fluorescence Recovery After Photobleaching (FRAP). Kinetic modeling of the FRAP data using a “reaction dominant” model yielded a FRAP residence time of ∼3-4 min in mES cells and somewhat longer in U2OS cells. How should we interpret this discrepancy? Both techniques have their limitations. SMT is limited by eventual photobleaching, whereas kinetic FRAP modeling is sensitive to anomalous diffusion, prone to overfitting and is an approximate technique as previously shown by Mazza *et al.*^62^ Moreover, since FRAP bleaches a large area (∼1–2 μm), local unbinding and re-binding (“hopping”^63^) can be mistaken for long individual binding events. Thus, we conservatively interpret the average CTCF residence time to be in the range of a few minutes – which is quite stable compared to conventional transcription factors (∼2-15 seconds),^62^ but very dynamic compared to the cell cycle (15-30 hours).

We next tracked the behavior of cohesin in live cells. After the onset of DNA replication in S-phase, cohesin has multiple roles other than TAD formation and maintenance such as sister chromatid cohesion and homologous recombination. Thus, since TADs are clearest in G1^54^ and the main function of cohesin in G1 is loop extrusion, we reasoned that cohesin dynamics in G1 should predominantly reflect the population of cohesin involved in chromatin looping. Surprisingly, FRAP and kinetic modeling revealed a G1 residence time of ∼20-25 min, ∼10-fold more stable than CTCF, which seemed inconsistent with the LMC model.

To explore this apparent discrepancy, we studied the diffusion dynamics of CTCF and cohesin. We developed an SMT technique to minimize experimental biases and a kinetic modeling framework^7^ (building on work by Mazza *et al.*;^62^ now available as a user-friendly web-interface called Spot-On^64^) and tracked single CTCF and cohesin molecules at 225 Hz. We found that CTCF and cohesin also exhibit very distinct diffusion dynamics: in mES cells, ∼50% of all CTCF molecules are bound to cognate sites. After unbinding, it takes a single CTCF molecule on average ∼1 min to find its next cognate site and the search mechanism involves large amounts of non-specific chromatin interactions (such as sliding or hopping; ∼35%) in addition to 3D diffusion (∼65%). In contrast, ∼40% of cohesin molecules are topologically engaged in G1; after unbinding, cohesin takes much longer (∼33 min) before topologically engaging chromatin and its search mechanism is dominated by 3D diffusion (∼77%). Finally, CTCF diffuses significantly faster than cohesin, which is inconsistent with them forming stable complexes in solution.

Thus, our imaging studies seemed incompatible with CTCF and cohesin forming a stable protein complex. To reconcile this, we performed super-resolution PALM and STORM imaging, localizing all single CTCF and cohesin molecules in a focal plane with a precision of ∼20 nm. We found that both CTCF and cohesin form small clusters – a property awaiting further investigation – and that these protein clusters tend to co-localize. Importantly, we found that a large fraction of CTCF and cohesin molecules indeed also co-localize at the single-molecule level inside cells.

**Figure 3.**
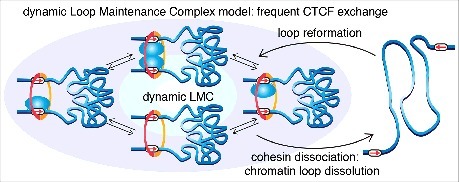
Dynamic LMC model. Cohesin functions to hold together two strands of chromatin and CTCF positions cohesin at its convergent cognate binding sites as previously proposed. Note that whether cohesin entraps DNA as a single ring or a pair of rings remains a subject of debate. In the dynamic LMC model, while cohesin holds together a chromatin loop, different CTCF molecules are frequently binding and unbinding, giving rise to a dynamic protein complex with a molecular stoichiometry that changes over time. Even though cohesin's average residence time (∼20–25 min) is much longer than CTCF's (∼1–4 min), cohesin does eventually dissociate. Since there is now nothing to hold together the chromatin loop, cohesin dissociation causes the loop to fall apart. Thus, we propose that TADs and the chromatin loops that hold them together are dynamic structures, and that CTCF and cohesin form a dynamic protein complex. This figure has been adapted and reproduced from Hansen *et al.*^7^

In summary, on the one hand our co-IP, ChIP-Seq and STORM studies strongly suggested that CTCF and cohesin form an LMC protein complex, whereas on the other hand our imaging studies showed that CTCF and cohesin cannot form stable complexes on chromatin or in solution. We reconcile these observations with a “dynamic LMC” model (Fig. 3). CTCF positions cohesin at loop anchors and cohesin holds together a chromatin loop as expected, but while cohesin holds together a chromatin loop, different CTCF molecules will unbind and rebind, giving rise to a dynamic LMC protein complex with a molecular stoichiometry that changes over time. Whether cohesin begins extruding again once CTCF has unbound or whether interaction with CTCF dissociates or inactivates the cohesin motor will be an important future question to be addressed. Since cohesin eventually does unbind (∼20-25 min residence time on average, which includes time to extrude a loop) at which point there is then nothing to hold the loop together, we propose that cohesin-mediated chromatin loops are dynamic structures that continuously form and fall apart throughout the cell cycle (Fig. 3).^7^ Thus, our results argue that TADs and chromatin loops, which are likely formed by cohesin-mediated loop extrusion, are dynamic structures with a mean lifetime of a few to tens of minutes in mES cells and most likely in most other actively dividing cell types (note that after cohesin dissociation, previously held-together chromatin will likely only move apart slowly even though the loop is broken; accordingly loops are likely more dynamic than TADs).

## Evidence that TADs and loops are dynamic from polymer simulation and acute CTCF/cohesin degradation studies

Polymer simulation studies provide a useful platform to test whether a mechanistic model can quantitatively account for experimentally observed Hi-C maps. Importantly, early polymer models suggested that a single stable loop induces frequent contacts near the base or anchor of the loop, but infrequent contacts between loci inside the loop, to an extent which is quantitatively inconsistent with Hi-C data.^65,66^ Thus, several polymer models did not predict stable loops.^38^ Moreover, the loop extrusion model introduced by Fudenberg and Imakaev *et al.*^8^ explicitly favored dynamic loops and convincingly argued against static loops. The best agreement with experimental Hi-C data^16^ was obtained when cohesin extrudes for ∼120-240 kb before falling off and a mean separation of ∼120 kb between extrusion complexes (note that these are average numbers).^8^ Crucially, in this model TADs emerge at the population level when averaged over the many distinct extrusion complexes at different positions in single cells (Fig. 2F). Moreover, this highly dynamic view is consistent with the relatively modest ∼2-fold insulation observed between loci in adjacent TADs.

Additional evidence for this view comes from recent Hi-C studies in *ΔWAPL* cells.^12,14,15^ Wapl removes cohesin from chromatin. Therefore, cohesin's residence time is prolonged in *ΔWAPL* cells,^12,14^ which – assuming no change in cohesin's extrusion speed – would increase cohesin extrusion processivity. If TADs were very stable structures, they should be largely unaffected in *ΔWAPL* cells, whereas if cohesin dynamics control TAD dynamics, TADs should be very sensitive to an increase in cohesin's residence time. Consistent with TADs being dynamic structures, the average TAD size increased substantially in *ΔWAPL* cells^12,14,15^ and the median loop size increased from 370 kb to 575 kb.^12^ We also note that these results are consistent with our imaging results showing that CTCF binding is stochastic and less stable than cohesin.^7^ If CTCF bound loop anchors more stably than cohesin or formed a very stable LMC with cohesin, increasing cohesin's residence time would not be expected to have a major effect on TAD size, since cohesin would be unable to extrude past the stable CTCF boundaries. Thus, the *ΔWAPL* Hi-C studies^12,14,15^ support our conclusion that CTCF binds more dynamically than cohesin.^7^

Further evidence for dynamic TADs come from recent studies employing auxin-AID mediated acute degradation of the cohesin subunit, Rad21.^13,14^ Upon auxin treatment, near-complete loss of Rad21 was achieved in 1 hour in Rao *et al.*^13^ or ∼20 min in Wutz *et al..*^14^ Rao *et al.* saw near-complete loss of loop domains after 40 min. Similarly, Wutz *et al.* found that 96% of loops, but only 53% of TADs were lost 15 min after auxin addition, whereas TADs were fully lost at later time points. These results show that loops and TADs rapidly disappear at a timescale of a few to tens of minutes after acute cohesin loss, which is consistent with the notion that cohesin binding dynamics control TAD dynamics. Moreover, in an informative experiment, Rao *et al.* degraded cohesin and then removed auxin to follow loop domain re-establishment by Hi-C as a function of cohesin replenishment. Loop domains were largely re-established 1 hour after auxin removal.^13^ Taken together, these studies^13,14^ suggest that the mechanisms by which TADs and loops are formed and broken down are both quite fast, which argues against stable TADs and favors dynamic TADs.

## Critical evaluation of evidence for TADs and loops being dynamic

We have proposed above that our imaging studies,^7^ recent polymer simulation studies^8^ and recent Hi-C studies employing acute cohesin degradation^13,14^ all point to TADs being dynamic. Here we will critically evaluate some limitations of each approach.

As mentioned, imaging benefits from directly observing CTCF and cohesin dynamics in live cells.^7^ However, imaging approaches also suffer from limitations. First, although the binding dynamics of single-molecules can be probed, it is not clear which locus they are bound to. For this reason, although we can report average residence times for CTCF (∼1–4 min) and cohesin (∼20-25 min in G1) in mESCs, these numbers are genomic averages. Almost certainly, some binding sites will exhibit longer and shorter binding times. Moreover, the binding times to cognate sites are highly stochastic and exponentially distributed such that few events exhibit the mean value. Second, by imaging it is very difficult to exclude that very small (<5%) subpopulations exist that exhibit much shorter or longer binding times. Third, although CTCF and cohesin both appear absolutely required for the formation of most TADs^10-15^ (∼18% of TAD boundaries were not sensitive to CTCF loss^10^ and could have different dynamics), using their binding dynamics to set a limit for the stability of TADs and loops is somewhat indirect. For example, if loops are held together by clusters of CTCF and cohesin, the dissociation of a single protein might not cause the loop to dissolve.

Likewise, polymer modeling suffers from a limitation that pertains to almost all modeling approaches: a large number of mutually inconsistent models tend to be able to fit the data. For example, recent polymer studies^8,9^ of presumably cohesin-motor driven loop extrusion were able to quantitatively account for Hi-C data, but so was a mechanistically distinct model by Benedetti et al., where supercoiling drives the formation of TADs.^66^ Furthermore, other polymer models could fit Hi-C data with different degrees of dynamics.^9,67^ Notably, a key advantage of dynamic loop extrusion models is that they can explain recent perturbation experiments where CTCF binding sites have been added or deleted^9,49-51^ and also explain the loss of either CTCF or cohesin.^10-15^ Nevertheless, it must be noted that the ability of a particular model to fit data does not prove that the model's mechanistic assumptions are also true.^68^

Finally, recent studies^13,14^ showing that TADs and loops are lost within tens of minutes after acute cohesin degradation and quickly reformed after cohesin replenishment, also have limitations. Although, they clearly show that TADs and loops can be formed and broken down quickly under these conditions, this does not necessarily mean that similar rates of turnover operate under normal conditions. Moreover, the time-resolution is relatively poor. For example, replenishment of cohesin involves translation of enough Rad21 protein, incorporation into cohesin complexes, loading onto chromatin, initiation and continuation of extrusion, and re-formation of TADs. Thus, although informative, it is difficult to reliably estimate hard numbers from such approaches.

## Conclusion and outlook

We have attempted to synthesize recent evidence from single-molecule imaging, polymer simulation and Hi-C approaches to suggest that most TADs and chromatin loops are very likely highly dynamic structures with a mean lifetime of a few minutes to tens of minutes in most actively dividing mammalian cells (genome organization dynamics could be different in some cell types, e.g. in post-mitotic olfactory sensory neurons, which exhibit a highly unusual and specialized nuclear organization^69,70^). Although each approach has its own advantages and limitations, we argue that in aggregate all the available evidence points to CTCF/cohesin-dependent TADs and chromatin loops being dynamic and not stable structures (note that since epigenomic compartments are formed by a distinct mechanism, whether compartments are stable or dynamic remains unknown). Taken together, these data are consistent with the view that TADs emerge at the population level when averaging over many dynamic and distinctly positioned loop extrusion complexes in many different cells (Fig. 2F).^8,9^ We note that dynamically extruding cohesin complexes are also likely to be functionally important by facilitating dynamic and repeated enhancer-promoter scanning in *cis.*

Nevertheless, a large number of questions remain unanswered. First, all the approaches mentioned above are somewhat indirect and final demonstration that TADs and chromatin loops are dynamic will require direct observation of specific loci in real-time in live-cells, for example by fluorescence labeling of the anchors of a loop. Defining the dynamics of individual TADs, understanding how they are controlled and differ from TAD to TAD and change during differentiation and gene activation will be an informative area for future studies. Second, it remains somewhat unclear how CTCF and cohesin interact. In particular, how can a ∼40-50 nm cohesin complex extrude extremely rapidly (∼10-50 kb/min) across chromatin, whilst at the same time being exquisitely sensitive to the orientation of a ∼3-5 nm CTCF protein? And how is cohesin extrusion powered and what happens once it encounters CTCF? Is the interaction direct^61^ or indirect?^71,72^ Perhaps using artificial roadblocks will enable defining which boundaries cohesin can and cannot pass *in vivo.* Third, whether cohesin forms a single ring, a dimer or oligomers remains a matter of debate.^35^ Conceptually, it seems more intuitive that a loop extrusion complex would be formed by a pair of cohesin rings, since this is more consistent with an ability to independently stop when each side reaches an occupied CTCF binding site, which is unlikely to occur simultaneously. But this is speculation and determining the structure and composition of putative extrusion complexes should be an urgent goal. Fourth, although high-resolution Hi-C^16^ and EM^73^ studies have defined the 3D organization of the genome in great detail, we still do not understand the 3D nuclear organization of the proteins, namely CTCF and cohesin, that control 3D genome organization. For example, we showed that both CTCF and cohesin form small protein clusters^7^ and a recent study found that CTCF can form large foci upon senescence, which causes genome reorganization.^74^ Thus, detailed studies on the nuclear organization of CTCF and cohesin should be informative. Fifth, absolute quantification of CTCF boundary permeability (i.e. fractional occupancy) and cohesin complex density on DNA will be essential to parameterize future polymer models. Finally, although TADs are somewhat conserved between different cell types,^1^ several do change. Thus, understanding the mechanistic basis for how TADs are regulated during differentiation, which likely involve additional proteins^75^ beyond CTCF and cohesin, will be crucial. This may one day enable therapeutic and controlled perturbations of 3D genome organization to be used in the treatment of disease.
